# Interferometric Sensor of Wavelength Detuning Using a Liquid Crystalline Polymer Waveplate

**DOI:** 10.3390/s16050633

**Published:** 2016-05-09

**Authors:** Paweł Wierzba

**Affiliations:** Department of Metrology and Optoelectronics, Faculty of Electronics, Telecommunications and Informatics, Gdańsk University of Technology, Narutowicza Street 11/12, 80-233 Gdańsk, Poland; pwierzba@eti.pg.gda.pl; Tel.: +48-58-347-2017

**Keywords:** polarization interferometry, waveplate, liquid crystalline polymer, tunable laser, two-beam interferometer

## Abstract

Operation of a polarization interferometer for measurement of the wavelength changes of a tunable semiconductor laser was investigated. A *λ*/8 waveplate made from liquid crystalline polymer is placed in one of interferometers’ arms in order to generate two output signals in quadrature. Wavelength was measured with resolution of 2 pm in the wavelength range 628–635 nm. Drift of the interferometer, measured in the period of 500 s, was 8 nm, which corresponded to the change in the wavelength of 1.3 pm. If needed, wavelength-dependent Heydemann correction can be used to expand the range of operation of such interferometer.

## 1. Introduction

Liquid crystalline polymers (LCPs) have been used to manufacture several innovative optical components, such as spiral phase plates, patterned polarizers and vortex retarders. The unique properties of these components have resulted in their use in numerous sensing and metrology areas, such as astronomy, polarimetry, and microscopy (*cf.* e.g., [1,2,3], respectively), enabling the development of new scientific instruments and measurement techniques. 

Polarization interferometry is also an area where components made from LCPs have a substantial application potential. A good example is the *λ*/8 waveplate, which can be used in polarization interferometers measuring displacement or wavelength, especially for operation in the UV-VIS spectral region, where zero-order crystalline waveplates are difficult to produce and expensive. 

A two-beam polarization interferometer using such a waveplate was designed for tracking changes in the wavelength emitted by an external cavity laser. The laser is a part of a measurement system that uses a technique similar to Swept-Source Optical Coherence Tomography (SS-OCT) to investigate properties of liquids. Operating in the wavelength range from 628 nm to 635 nm, the laser provides information about the instantaneous value of the wavelength with uncertainty better than 0.1 nm. Consequently, the designed interferometer should have the measurement range of at least 0.1 nm, and resolution about 2 pm. Since the measurement does not last more than 6 s, the long-term drift of the interferometer is not critical. 

**Figure 1:**
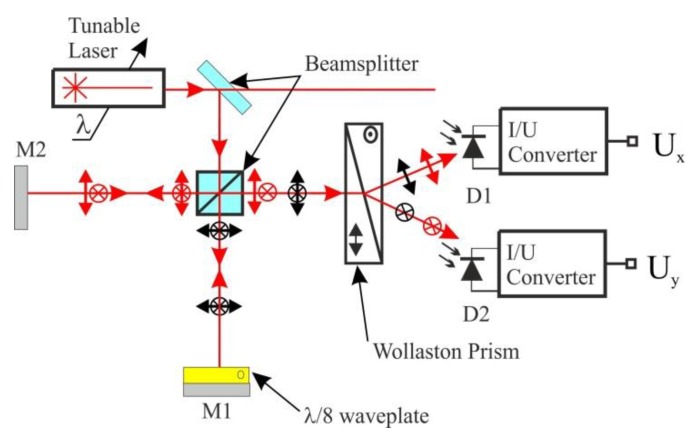
Polarization interferometer for measurement of change in the wavelength of a tunable semiconductor laser. D1, D2—photodiodes, M1, M2—mirrors, *U*_x_, *U*_y_—output voltages.

The interferometer, whose layout is shown in Figure 1, was designed as a modification of the interferometers described in [4]. A *λ*/8 waveplate made from LCP, rather than two total internal reflections, is used to obtain a π/4 phase shift between the two orthogonally polarized components of the beam propagating in one arm of the interferometer.

Innovative character of the presented interferometer is in adapting a metrology-grade solution [4] and implementing it with an LCP component in a way that allows competition with other high-resolution wavelength measurement solutions.

Compared to the solutions based on tunable Fabry-Perot interferometers, presented interferometer is more versatile, as it does not use moving parts which limit the measurement speed and has measurement range that can be adjusted by changing the optical path difference. Its output is a pair of analog signals that are available instantaneously and provide information about the wavelength detuning with little additional processing. Compared to the solutions based on Fizeau interferometers, in which the wavelength is determined from the fringe pattern, presented setup is faster, as it does not use a camera or a CCD linear array which limiting the measurement rate and does not rely on computationally intensive signal processing methods that introduce delays in the measurement process.

## 2. Materials and Methods

### 2.1. Operation of the Interferometer

Consider operation of an interferometer presented in Figure 1. Let us assume that it is illuminated with a monochromatic beam of intensity *I*_0_, either circularly polarized or linearly polarized with the direction of polarization making an angle of 45° or −45° with the plane of the figure. The intensity of the light incident on detectors D1 and D2 can be expressed as:

(1) $$\begin{array}{l} I_{1}=\frac{I_{0}}{4}[1+\cos\phi ]\\ I_{2}=\frac{I_{0}}{4}[1+\sin\phi ] \end{array}$$

where *φ*—phase difference of interfering beams, given by:

(2) $$\phi =\frac{2\pi }{\lambda }\;\Delta l$$

where *λ*—wavelength of the beam, Δ*l*—optical path length difference between the two arms of the interferometer. By changing Δ*l*, the measurement range of the wavelength Δ*λ* can be adjusted.

The optical path length difference Δ*l* corresponding to required Δ*λ* can be obtained from Equation (2) by setting the change of phase *φ* corresponding to Δ*λ* equal to 2π, *i.e.*:

(3) $$\Delta \phi =2\pi =\frac{2\pi }{\lambda_{C}}\;\Delta l-\frac{2\pi }{\lambda_{C}+\Delta \lambda }\;\Delta l$$

where Δ*φ*—change of phase, *λ*_C_—central wavelength of operating range of the interferometer, Δ*l*—optical path length difference, Δ*λ*—measurement range of the wavelength. For *λ*_C_ = 633.5 nm and Δ*λ* = 0.1 nm, Δ*l* equals 3.99 mm.

### 2.2. Manufacturing of the λ/8 Waveplate

**Figure 2:**
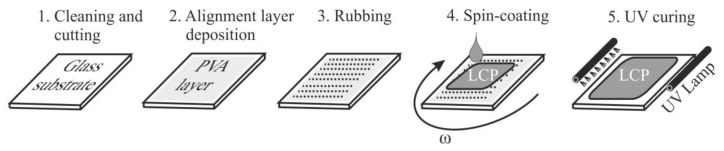
Manufacturing process flow of the LCP waveplate.

The LCP waveplates were manufactured using technology essentially identical to that described in detail in [5]. Two minor modifications were introduced in the process shown schematically in Figure 2. First, the heating step between spin-coating and UV curing was removed, thereby eliminating virtually all point- and bubble-like defects and discontinuities of the LCP surface. Instead, a gentle flow of warm air (25–35 °C) was induced over the surface of the LCP layer before and during curing to promote the solvent evaporation. Second, a high power UV LED whose central wavelength was 385 nm was used as the UV curing lamp.

A series of several LCP waveplates were made on substrates from microscopy glass cut to 25 mm by 25 mm size. The quality of most LCP layers was good, virtually without defects in the central part of the waveplate. Similarly, the retardation in the central part of the waveplate varied by less than 5% of the mean value. Overall, the optical quality of the waveplates is fairly good. The only exceptions are the corner areas, where the LCP layer becomes thicker and its alignment is not as good as in the central part of the waveplate.

It should be noted that in commercially produced equipment the waveplate can be deposited either on the beamsplitter face or on the mirror surface, thereby reducing the cost and complexity of the optomechnical assembly as well as facilitating the alignment of the optical setup.

The process parameters, such as rotation speed and the amount of solvent were selected, based on our previous experience, to yield waveplates with π/8 retardation at 633 nm. Retardation of the waveplates was measured using a He-Ne laser and a PAX5710VIS polarimeter (Thorlabs, Newton, NJ, USA). A waveplate whose retardation was within 2% of the nominal π/8 value was selected for use in the interferometer.

### 2.3. Detection Electronics

**Figure 3:**
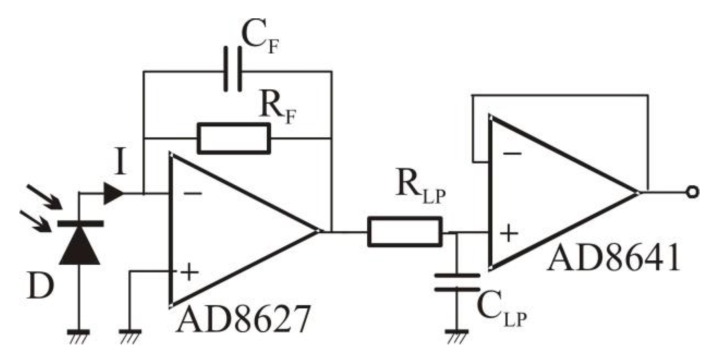
Detection electronics (one channel of two). D—photodiode. Further explanations in the text.

The detection electronics consisted of two identical channels, schematically presented in Figure 3. Each channel comprises a photodiode (BPW34, Osram Opto Semiconductors, Regensburg, Germany) followed by transimpedance amplifier (current-to-voltage converter), a low-pass filter, consisting of resistor *R*_LP_ and capacitor *C*_LP_, and a buffer. The transimpedance amplifier, built using an operational amplifier (AD8627, Analog Devices, Norwood, MA, USA), converts the current *I* from the photodiode into voltage *U* according to:

*U* = *R*_F_·*I* (4)

where *R*_F_—transimpedance (*R*_F_ = 100 kΩ). Capacitor *C*_F_ improves stability of the transimpedance amplifier. The low-pass filter, with cut-off frequency of 10 kHz, filters out the broadband noise. The filter is followed by a buffer (AD8641, Analog Devices) driving a sixteen-bit data acquisition card (NI-9215, National Instruments, Austin, TX, USA) or an oscilloscope (TDS3032, Tektronix, Beaverton, OR, USA).

The detection electronics was built on a printed circuit board. It was powered by a ±12 V stabilized laboratory power supply.

### 2.4. Setup and Alignment

**Figure 4:**
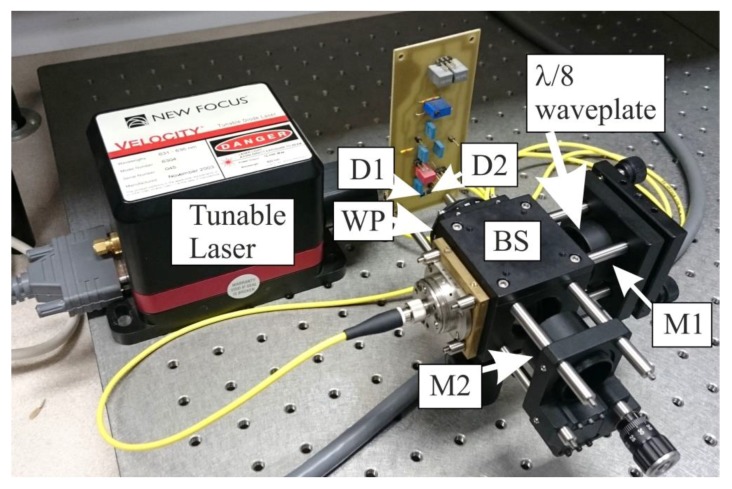
Polarization interferometer during the alignment procedure. M1, M2—mirrors; BS—beamsplitter; WP—Wollaston prism; D1, D2—photodiodes.

The interferometer was built using off-the-shelf optical and optomechanical components and mounted on an optical plate. A cube beamsplitter BS was used to reduce the influence of dispersion by ensuring that the beams in both arms propagate through the same distance in glass. For alignment, a 6304 tunable laser (New Focus, Santa Clara, CA, USA) was connected to the input of the interferometer via a collimator, as shown in Figure 4.

The alignment comprised three steps. First, the mirrors were aligned so that interference signal was obtained at detectors D1 and D2. In the second step, the optical paths in both arms were made equal. In this step the initial setting was performed using a dial caliper, followed by precision adjustment done by the micrometer actuator in the mount of mirror M2. During the latter phase the laser was tuned in the range of 2 nm to detect the position of mirror M2 in which the output signal remained constant. In the third step, the mount with mirror M2 was pushed forward by a differential micrometer head, by 1.995 mm, in order to introduce the optical path length difference Δ*l* = 3.99 mm.

When the alignment has been completed, the measurements were performed. First, the stability of amplitude and wavelength of the New Focus 6304 tunable laser was investigated. The wavelength of the laser was set constant at 635 nm and emitted power was set at 1.6 mW. The power emitted by the laser was measured by a 1835-C optical power meter (Newport, Irvine, CA, USA). Wavelength stability of the laser was tested by comparison with a scanning Fabry-Perot interferometer (SA-210, Thorlabs) offering the Free Spectral Range of 10 GHz (13.5 pm at 635 nm) and the resolution of 67 MHz (0.90 pm at 635 nm). Second, optical path length difference Δ*l* was set to zero and drift of the interferometer was investigated. The voltages *U*_x_ and *U*_y_ at the outputs of the two channels of detection electronics (*cf.* Figure 1) were recorded as a function of time. Following, the phase *φ*(t) was calculated based on the values of *U*_x_ and *U*_y_. Knowing *φ*(t) it was possible to calculate the drift of optical path length difference Δ*l*, using Equation (2), as shown in Section 3.2. Finally, the wavelength emitted by the laser was measured using the interferometer. 

## 3. Results

First, results of drift measurement of the laser are presented in Section 3.1. Second, results of drift measurement of the interferometer are presented in Section 3.2. Finally, the response of the interferometer to the wavelength change is presented in Section 3.3.

### 3.1. Stability of the Laser

**Figure 5:**
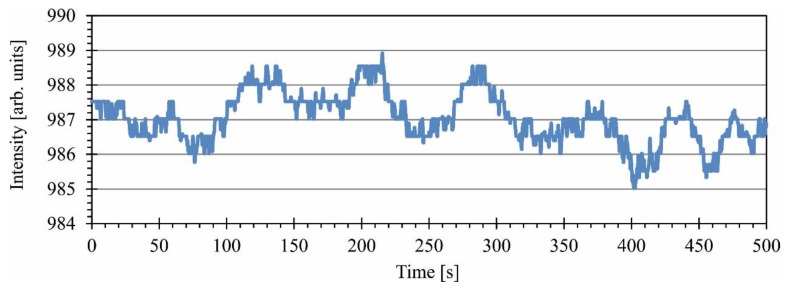
Drift of the optical power emitted by the tunable laser.

In general, stability of the laser depended on the wavelength, emitted power level, ambient temperature and the time from powering up. Example results, obtained for operation at 635 nm with 1.6 mW output power are presented in Figure 5.

In general, the intensity was stable to within ±1% of emitted power. The drift of the wavelength was 4.3 pm peak-to-peak in a measurement lasting 500 s.

### 3.2. Drift Measurement

A small shift of phase difference of interfering beams *φ*, can be expressed, based on Equation (2) as:

(5) $$d\phi =\frac{2\pi }{\lambda }\;d(\Delta l)-\frac{2\pi }{\lambda^{2}}\Delta l\;d\lambda \;$$

where d*λ*—change of the wavelength, d(Δ*l*)—change of optical path length difference between the two arms of the interferometer. When Δ*l* = 0, Equation (5) becomes:

(6) $$d\phi =\frac{2\pi }{\lambda }\;d(\Delta l)$$

reducing the impact of instability of the laser source wavelength d*λ* on the measurement of the phase change d*φ*.

**Figure 6:**
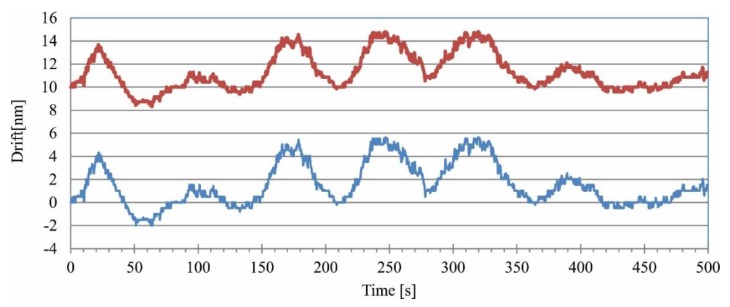
Drift of the interferometer. Upper trace—drift before applying the Heydemann correction, lower trace—drift after applying the Heydemann correction.

From Equations (5) and (6) follows, that performing measurements of the phase change d*φ* when Δ*l* = 0, makes the measurement largely independent of instability of the laser source wavelength d*λ*. Therefore, measurements of phase were performed in normal laboratory conditions for Δ*l* = 0, and the Heydemann correction [6] was applied to them. The results before and after applying the correction are shown in Figure 6 (top and bottom trace, respectively). The interferometer was adjusted in such a way that the change introduced by the Heydemann correction was the largest—amplitude of the drift was increased by about 20% and the 10 nm shift was applied.

As can be seen from Figure 6, the peak-to-peak drift after correction is 8 nm. This value will be used later to estimate the error introduced by this drift to the measurement of wavelength.

### 3.3. Wavelength Measurement

**Figure 7:**
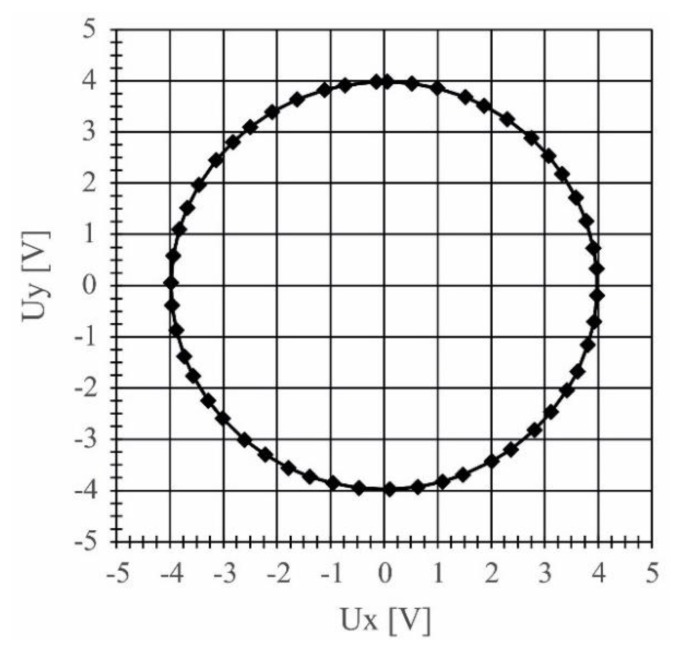
Output signals *U*_x_ and *U*_y_ from the interferometer after removal of the constant components and application of the Heydemann correction.

The wavelength measurement was performed step-by-step, by manually tuning the laser, using the piezoelectric fine tuning feature, to required frequency, measured by the scanning Fabry-Perot SA-210 interferometer. When the required frequency was attained, the output of the interferometer (*i.e.*, the signals *U*_x_ and *U*_y_, corresponding to intensity *I*1 and *I*2) was recorded. Resolution of such measurement was better than 0.4 pm. The Heydemann correction was applied, and signals *U*_x_ and *U*_y_ after correction, corresponding to the wavelength change of 0.1 nm, are shown in Figure 7.

Based on the acquired data, and taking into account the noise of the current-to-voltage converters, it can be concluded, that the required resolution of 0.002 nm was attained. Determination of accuracy is much more difficult, as it would require comparison with a wavelength meter, not available to this author.

Stability of the interferometer, defined as the wavelength error *δλ* resulting from drift in *δ*Δ*l* can be calculated from Equation (2), by writing:

(7) $$\frac{2\pi }{\lambda }\;\Delta l=\frac{2\pi }{\lambda +\delta \lambda }\;\left(\delta \Delta l\right)$$

When *λ* = 635 nm, Δ*l* = 3.99 mm, *δ*Δ*l* = 8 nm, the wavelength error *δλ* = 1.3 pm. Stability of the interferometer can be improved by using techniques such as vibration isolation, airflow restriction, temperature control and operation of the interferometer in an evacuated environment.

## 4. Discussion

A drawback of presented interferometer is the dependence of the phase change introduced by the *λ*/8 waveplate on the wavelength. In this particular case, the maximum change in the wavelength is 3.5 nm from its central value. In the absence of LCP birefringence dispersion, this would correspond to the phase change *δλ* of 8.7 mrad, *i.e.*, 0.50°. In the target application the error introduced by this *δλ* is acceptable, and any correction is not needed.

However, it should be noted that such correction is possible when better accuracy or a broader range of operating wavelength is needed. In such a case a wavelength-dependent Heydemann correction could be used. In this method the correcting parameters are calculated in advance as a function of the wavelength and applied later to the *U*_x_ and *U*_y_ signals. Values of these parameters are selected, based on the instantaneous value of the laser wavelength. 

Assuming that the error caused by phase change *δλ* less than 87 mrad (*i.e.*, 5°) can be corrected by wavelength-dependent Heydemann correction, the operating wavelength range would reach ±35 nm around the central value. In practice, it will be smaller due to the dispersion of the LCP birefringence that must also be taken into account.

## 5. Conclusions

A polarization interferometer using a *λ*/8 waveplate made from liquid crystalline polymer was demonstrated. This two-beam interferometer was designed to measure small changes in the wavelength of optical radiation emitted by an external cavity laser operating in the range from 628 nm to 635 nm. Measurement resolution of 2 pm was attained. Stability of the interferometer, tested in the period of 500 s, was about 1.3 pm. The wavelength range of the interferometer can be extended by applying the wavelength-dependent Heydemann correction to the output signals. The *λ*/8 waveplate can be deposited on the beamsplitter face or on the mirror surface, reducing the cost and complexity of the optomechnical assembly. Such interferometer can be used in applications where high resolution wavelength measurement is needed, e.g., in optical fiber sensing, especially in Fiber Bragg Grating sensors or Fabry-Perot sensors [7].
